# Emergency Medical Service Providers' Perception of Health-Threatening Stressors in Emergency Missions: A qualitative Study

**DOI:** 10.4314/ejhs.v31i3.8

**Published:** 2021-05

**Authors:** Ali Afshari, Seyed Reza Borzou, Farshid Shamsaei, Eesa Mohammadi, Leili Tapak

**Affiliations:** 1 Nursing and Midwifery School, Hamadan University of Medical Sciences, Hamadan, Iran; 2 Chronic Diseases (Home Care) Research Center, Hamadan University of Medical Sciences, Hamadan, Iran; 3 Maternal and Child Care Research Center, Behavioral Disorders and Substance Abuse Research Center, Hamadan University of Medical Sciences, Hamadan, Iran; 4 Faculty of Medical Sciences, Nursing Department, Tarbiat Modares University, Tehran, Iran; 5 Department of Biostatistics, School of Public Health, Modeling of Noncommunicable diseases Research Center, Hamadan University of Medical Sciences, Hamadan, Iran

**Keywords:** stress, emergency medical service, qualitative study

## Abstract

**Background:**

Unknown and unpredictable situations cause emergency medical service (EMS) providers to experience various stressful factors. These factors are affected by sociocultural conditions and expectations of the casualty and affect EMS providers' performance and health at the incident scene. The present study was conducted to explore EMS providers' perception of stressful and health-threatening factors in emergency missions.

**Method:**

This qualitative conventional content analysis was conducted in 2020. The participants included 16 EMS providers working at the Emergency Medical Services Department in Hamadan Province, Iran. The participants were selected using purposive sampling and underwent semi-structured interviews until data saturation. Data were analyzed using the Graneheim and Lundman method.

**Results:**

Analysis of the interview data yielded six subcategories (i.e., incident scene hazards, violence-related injuries, physical injuries caused by patient care/handling, ambulance crash-related injuries, emotional impact of patients' suffering and ailments, and highly stressful missions), two main categories (i.e., physical injuries and psychological tensions), and a theme of occupational injuries.

**Conclusions:**

According to the results, in addition to having concerns about caring for patients and saving the injured, EMS providers also worry about potential threats to their own health. The present study identified and described some major stressors in emergency missions. Thus, for a better and more effective efficiency, the present study results can be used to reduce or modify stressors in EMS providers.

## Introduction

Emergency medical services (EMSs) have a major role in providing patient care in prehospital settings and also in providing medical services throughout the world (1). One of EMS providers' responsibilities is to assess and stabilize the patient before receiving hospital services (2). As a major group of healthcare specialists, EMS providers prepare first aids and care for patients and the injured, transport patients, and record operations, and, in this way, they often experience difficult and dangerous situations like severe accidents or life-threatening acute diseases (3). EMS providers make quick decisions and perform procedures in complex situations (4). Therefore, they are exposed to many unpredictable threats and stressors during emergency missions. EMS providers experience a lot of stress in their workplace (5), such that 22% of them are affected by stress complications (6). The frequency and repeatability of exposure to critical accidents can cause psychological complications such as PTSD in EMS providers (7). Some consequences of stress in EMS providers include anxiety and distress (8), low job satisfaction (9), increased care errors, ambulance traffic accidents, PTSD, mental health disorders, irritability, and headache (10).

Some quantitative studies have identified stressors in EMS providers using various questionnaires, including being exposed to dangerous environments and performing emergency responses (11), performing care in uncontrollable conditions (12), being far from resources, attending crime scenes(13), experiencing violence and threats (14), and being exposed to frightening scenes such as violence, suicide, and death (15). Some of these factors predispose EMS providers to certain health threats in the short- or long-term (16). Thus, providing safe conditions for EMS providers during emergency missions is a vital principle to ensure safety in this profession.

Occupational stress has had an increasing trend in recent years, and has turned into a persistent global issue. Stress is a complex phenomenon that is present in most healthcare systems (17). Although people's characteristics significantly affect the stress level, the context of stressors has a major role in causing stress. Thus, type and severity of stress vary from one setting to another depending on the culture, work environment, interpersonal relationships, and social interactions (18). Some quantitative studies have been conducted on occupational stress in EMS providers in Iran (19–21). However, searching databases yielded no published qualitative research on EMS providers' perceptions of stressors in emergency missions. Thus, the present study was conducted to explore EMS providers' perception of health-threatening stressors in emergency missions.

## Materials and Methods

**Study design and setting**: This qualitative study was conducted using the qualitative approach and the conventional content analysis method. Content analysis is an analytical approach that provides insights into understanding the phenomenon under study (22). The present study was conducted between December 2019 and April 2020 in emergency medical bases in Hamadan province. All EMS providers were male, with academic qualifications such as associate's degree and bachelor's degree in EMS as well as bachelor's degree and master's degree in nursing.

**Participants:** The study population included the operational personnel in urban and rural emergency bases affiliated to Hamadan's emergency medical system. The study inclusion criteria were a minimum of two years' professional experience in emergency medical departments and willingness to take part. The participants were selected using purposive sampling. In the present study, the data reached saturation after interviewing 12 participants, but four more interviews were conducted for greater certainty. A total of 16 EMS providers participated in the study.

**Data collection:** Data were collected using semi-structured face-to-face. The first author conducted the interviews (each lasting 45–60 minutes) outside the working hours with prior arrangement with the participants. The initial questions were open-ended: “Describe your experience of the job you are in”, and “Could you talk about some emergency missions you were dispatched to?” The next questions were asked according to the participants' narratives and the study objectives: “Have you ever been under pressure in emergency missions?”, “Could you talk about these situations?”, and “How did you feel in these situations. Each interview was recorded with an MP3 player and then transcribed verbatim by the first author.

**Data analysis:** The data were analyzed according to the Graneheim and Lundman five-step method (23). First, the interviews were transcribed, typed in Microsoft Word®, and read several times to extract meaning units. Then, the interview data were continuously compared, the relationship between concepts was determined, and the initial codes were classified according to their similarities and differences. Next, categories with common meanings were integrated in separate groups, and finally, the main themes were extracted according to meanings and the relationship between classes. This research was part of a PhD dissertation supported by the Hamadan University of Medical Sciences.

**Data rigor:** First, two pilot interviews were conducted to identify the proper form of interview and questions. Credibility was ensured by the researchers' profound and prolonged engagement with the data as well as by member checking. Accordingly, after the extraction of the initial codes, the transcripts were made available to the participants to correct or confirm the initial codes. Moreover, confirmability was ensured by peer checking, as some of the interviews along with the extracted codes and categories were presented to two experts in qualitative studies (outside the study team) to confirm the codes. Further, dependability was ensured as the three study team members with excellent experience in qualitative studies separately performed all encoding stages and reached consensus after discussing analysis results for several sessions. Finally, diversity of the samples, description of their conditions, and comparison of the results with those of other studies helped transferability of the data.

**Ethical considerations**: The present study was approved by the Ethics Committee of the Hamadan University of Medical Sciences (code: IR.UMSHA.REC.1398.685). The participants took part in the study on a voluntary basis. Before the interviews, the study objectives were explained first verbally and then in writing. The participants signed the informed consent form, allowing recording of interviews and anonymous use of data, and were assured that they could withdraw at any stage of the study.

## Results

There were 16 participants in the study with a mean age of 36 years, ranging from 26 to 50 years and an average work experience of 14 years. Other characteristics of the participants are reported in Table 1.

**Table 1:** Characteristics of the participants

Characteristics of Participants	N (%) or Range (Median)
Educational degree	
EMT	4(25)
BS in EMS	3(19)
BS in Nursing	4(25)
MSc in Nursing	5(31)
Years in Practice	4 – 28(14)
Age	26 – 50(36)
Gender	
Male	16(100)
Marital Status	
Married	12(75)
Single	4(25)
Ambulance station location	
Urban	10(62)
Rural	6(38)

The analysis, integration, categorization, and organization of the extracted codes and subcategories based on their similarities and differences led to extraction of two main categories and one theme. The main categories included physical injuries and psychological tensions, and the central theme was “occupational injuries” (Table 2).

**Table 2:** Main categories and subcategories of health-threatening stressors in emergency missions

Theme	Main categories	Subcategories
Occupational injuries	Physical injuries	Incident scene hazards
		Violence-related injuries
		Physical injuries caused by Patient care/handling
		ambulance crash-related injuries
	Psychological tensions	emotional impact of patients' suffering and ailments
		highly stressful missions

**Occupational injuries**

**1. Physical injuries**

Physical injuries can occur in some missions for various reasons, including unknown circumstances of emergency missions, as cited by most of the participants. The category of physical injuries consisted of four subcategories: “incident scene hazards”, “violence-related injuries”, “physical injuries caused by patient care/handling”, and “ambulance crash-related injuries.”

1.1. Incident scene hazards

The participants stated that they had suffered certain physical injuries in inherently complex and high-risk scenes such as scenes with hazardous materials or unstable cars present, or due to being dispatched to missions in unfamiliar locations such as outskirts of cities and off the main roads:

“*We were dispatched to a warehouse of chemicals to rescue one of the workers …. By the end of the mission, we had fairly severe respiratory symptoms, for which, we were kept under observation in a hospital for a few hours*.” (P6)

1.2. Violence-related injuries

Despite efforts made by EMS providers to reach the accident scene as soon as possible, they are subjected to violence and threats by the patient or their family in some emergency missions. The participants explained that these behaviors were mostly due to anger of the patient's family caused by the severity of the condition or death of the patient and the misplaced expectations of their family or people gathering around the accident scene:

“*In a road accident, the brain of the casualty had been smashed up and he had died instantaneously. Despite arriving on the scene without delay in less than six minutes, we were attacked and physically injured by his relatives.*” (P9)

1.3. Physical injuries caused by patient care/handling

Due to the nature of this profession as the first line of exposure to diseases or road accidents, EMS providers are exposed to many infections transmitted through breathing or the patient's moist body substances. Furthermore, one of the main and routine duties of EMS providers in all missions is lifting and handling patients and heavy emergency equipment, which expose them to musculoskeletal injuries and cause development of certain diseases:

“*The patient was in a critical condition, and we had to quickly transfer him with a scoop. Due to his heavy weight and the narrow staircase, I suffered a twisted knee. A week later, MRI revealed torn cruciate ligament, for which, and I had to undergo ligament repair surgery*.” (P2)

1.4. Ambulance crash-related injuries

Driving the ambulance is part of EMS providers' daily tasks. Most of the participants mentioned that they strongly felt the danger of traffic accidents when transporting seriously ill and injured patients from the scene to trauma centers. Some of the participants who had experienced traffic accidents while driving the ambulance talked about severe stresses following physical injuries caused by such accidents.

“*On the way to transfer a patient when it was raining and the roads were slippery, we smashed into a car in front … the ambulance's front bumper and the body were badly mangled, and my colleague's wrist hit the equipment in the backspace of an ambulance and broke*.” (P11)

**2. Psychological tensions**

One of the main factors disturbing mental health in EMS providers is psychological tensions caused by the direct exposure to a variety of injuries and intensely stressful scenes as well as frequent exposure to grieving patients and families. The main category of psychological tensions included two subcategories: “emotional impact of patients' suffering and ailments” and “highly stressful missions.”

2.1. Emotional impact of patients' suffering and ailments

In their interviews, the participants pointed out that their mood changed after several years of working in this profession due to the stress in most missions and frequent and daily exposure to people's ailments and suffering. The participants even talked about the effect of their altered moods on their family relationships:

“*I don't remember at all the last time I laughed whole-heartedly. The missions' stresses and the patients' suffering have affected us so much that I'm not in mood at all to joke and laugh with my kid. I used to be a happy person, and I know I am no longer like before*.” (P7)

2.2. Highly stressful missions

The participants mentioned exposure to intensely horrific scenes in some missions such as scenes related to burns, building collapses, and explosion scenes. They argued that although these missions were few and far in between, they were extremely troublesome and stressful, and their effects would persist for many years in one's thoughts, behavior, and private life.

“*In one of these missions, four passengers had been trapped in a burning car. I witnessed them burn. … I was badly stressed for several months and experienced nausea and vomiting at meal times. Even after two years, I still have nightmares about that scene some nights.”* (P4)

## Discussion

Based on the present study's objectives, stressors experienced by the EMS providers in emergency missions, associated with health threats were classified into two main categories: “physical injuries” and “psychological tensions.” Most of the participants referred to physical injuries in emergency missions as one of the main problems and stressors of their job, such that some of them had suffered severe injuries on the scene. EMS providers, in most of their emergency missions, provide relief in uncontrolled scenes, without being aware of potential risks (12). Being exposed to hazardous environments and inherently high-risk processes in emergency missions can cause certain injuries to EMS providers (24). In studies conducted in the US and some other countries, environmental risks of the scene included sharp objects, contact with chemicals, fall from height, and electrocution (25–27).

Recent studies have shown that violence and threat against EMS providers have exacerbated both verbal threats and physical aggressions during their missions (28, 29). Petzall et al. reported that 66% of ambulance personnel in Sweden experienced threats or violence, with physical aggression being its most common form (30). Other studies have shown that violence is a huge concern against EMS personnel on duty, such that 87.5% of them experience various cases of violence that mostly cause of injuries (14, 31). One of the main reasons for violence and aggression against EMS providers is that they are in the frontline of facing people and patients (32). Another reason for violence can be the expectations of patient's relatives to provide services in the shortest time, while they are highly anxious and not in a normal state.

The participants also referred to physical injuries suffered while providing care and handling patients. Most of the participants described their experiences about musculoskeletal injuries and development of certain diseases. They mentioned the following factors in this regard: inaccurate knowledge of the patient condition and history of diseases, the defective process of disinfection in missions due to time limitations, and lack of personal protective equipment. Thomas et al. reported that although transmission of infection to EMS providers was descending, the risk was significant during the certain respiratory disease epidemic (12). Although EMS providers use personal protective equipment on missions, one of the reasons for transmission of infections is the uncontrolled and unfamiliar environment of the scene and people's disease. Another finding in the present study was musculoskeletal injuries caused by activities in emergency missions, patient lifting and handling ambulance equipment. In this regard, the participants mentioned factors such as not observing ergonomic principles due to the scene conditions, lifting heavy patients, handling some heavy equipment, and performing care in unstable conditions in the backspace of an ambulance. Over 63% of work-related accidents in EMS providers are caused by excessive pressure or changing physical positions, and the most common conditions that cause injury include working in emergency situations, carrying patients, and carrying stretchers (2). Punnett and Wegman reported that transferring patients exacerbating abnormal body postures and the impact of strong forces were the two significant factors causing musculoskeletal injuries (33). A study by Wang et al. showed that 14% of all physical injuries occurred during stretcher operations while moving the stretcher loaded with a patient (34).

Another finding of the present study was injuries caused by ambulance accidents. The majority of the participants, especially those with shorter work experience, talked about ambulancerelated accidents and mentioned factors such as driving at night under unfavorable road conditions. Driving the ambulance is one of the daily tasks of EMS providers, which exposes them to many driving-related risks. The multiple consequences of ambulance accidents include EMS providers' injury, delay in rescue, increased costs for the individual and the organization, and reducing patient's chance of survival (35, 36). Studies have shown that driving under emergency conditions compared to non-emergency conditions increases the risk of accidents two-fold, serious injuries eight-fold, and car and equipment damage 17 fold. Moreover, driving under emergency conditions can also significantly increase the risk of accidents even when driving with the siren on (37, 38).

The present study results showed that being on emergency missions, providing care and empathy with patients, and in some cases, being exposed to heartbreaking scenes can impair the mental health of EMS providers. The participants stated that they had suffered symptoms like boredom, lack of focus, and anxiety because of stresses on the scene and direct contact with highly stressful scenes. A study by Lepiesza and Binczycka-Anholcer showed that 19% of EMS providers suffered uncontrollable episodes of anger, shouting, and other negative emotional behaviors following working stress in prehospital settings (14). Frequent exposure to intensely stressful scenes can cause mental disorders, especially PTSD in this group of health cares (39). Some of these stressful events include witnessing death, people being subjected to physical violence, shooting scenes, and serious car accidents (40). Studies have shown that 82% of EMS providers have experienced some of these events, and 9% to 22% of them are estimated to have PTSD symptoms (16). In the end, the results of this study showed that despite the numerous stressors in emergency missions, EMS providers save the lives of patients and the injured by sacrificing and risking their health in emergencies.

The present study results suggest that EMS providers suffer from physical and psychological stressors on emergency missions. These factors threaten EMS providers ‘health and are regarded as one of their concerns. We identified some of the stressors in this group, some of which are preventable and some are work-related. EMS managers and policymakers can reduce or modify some stressors in this field with planning and implementing measures such as execution of stress management courses, the presence of psychologists advisors in the emergency medical system, and execution inter-professional meeting.
